# Synergistic pairing of *Lactobacillus helveticus* Stellar Lh-170 and *Lactiplantibacillus plantarum* Stellar Lp-199 suppresses *Streptococcus mutans* and disrupts cariogenic biofilms

**DOI:** 10.1080/20002297.2026.2723648

**Published:** 2026-09-29

**Authors:** Kris M. James, Zack Klassen, Peter A. Cadieux, Jeremy P. Burton, Maxim D. Seferovic

**Affiliations:** a Canadian Centre for Human Microbiome and Probiotic Research, Lawson Research Institute, London, Ontario, Canada; b School of Nursing, Fanshawe College, London, Ontario, Canada; c Stellar Biome, London, Ontario, Canada; d School of Applied Science and Technology, Fanshawe College, London, Ontario, Canada; e Department of Microbiology and Immunology, The University of Western Ontario, London, Ontario, Canada; f Department of Surgery, The University of Western Ontario, London, Ontario, Canada; g Department of Obstetrics and Gynecology, Baylor College of Medicine, Houston, TX, USA; h Department of Obstetrics and Gynecology, Texas Children’s Hospital, Houston, TX, USA

**Keywords:** *Lactobacillus*, *Lactiplantibacillus*, probiotics, dental caries, *Streptococcus mutans*, biofilm

## Abstract

**Background:**

*Streptococcus mutans* exists within a biofilm ecosystem where neighboring microbes influence each other’s viability. Probiotic strains with complementary antagonistic properties may enhance suppression beyond individual strains. An evaluation of probiotic combinations assessed synergistic biofilm and pathogen suppression.

**Methods:**

Nineteen probiotic strains were screened for hydrogen peroxide production and antagonism of *Streptococcus mutans*; five candidates were further tested in two-strain combinations for synergistic antagonism of *S. mutans* and hydroxyapatite adhesion. An artificial saliva culture model assessed both the inhibition and disruption of *S. mutans* biofilms alone and with *Candida albicans*, with findings validated on human molars.

**Results:**

Of the 19 strains assessed, *Lactobacillus helveticus* Stellar^TM^ Lh-170 and *Lactiplantibacillus plantarum* Stellar^TM^ Lp-199 exhibited the strongest synergistic antagonism against all tested *S. mutans* strains (*p* < 0.001). Both strains rapidly produced H_2_O_2_ and adhered to hydroxyapatite. When co-incubated at a 10:1 ratio with S*. mutans*, the combination reduced biofilm biomass by 62% (n.s.) and significantly reduced *S. mutans* viability below detectable CFU limits (~10^7^-fold reduction). When co-incubated with *S. mutans* and *C. albicans* together, dose-dependent reductions in the viability of both pathogens were observed. When pre-established *S. mutans* biofilms were treated with the two-strain combination, the number of viable *S. mutans* CFUs was reduced by >  10,000-fold *in vitro* and 3,135-fold in extracted human molars after 24 h (*p* < 0.0001).

**Conclusions:**

Synergistic strain combinations effectively modulate cariogenic pathogens *in vitro, exceeding benchmark strains*. These findings support the clinical evaluation of the *Lactobacillus helveticus* Stellar Lh-170 and *Lactiplantibacillus plantarum* Stellar Lp-199 combination for prophylactic caries risk management.

## KEY MESSAGES

*Lactobacillus helveticus* Stellar Lh-170 and *Lactiplantibacillus plantarum* Stellar Lp-199 synergistically inhibit *S. mutans* growth and biofilm formation.

## Introduction

Dental caries begins with enamel demineralization and, if untreated, progresses irreversibly into the dentin, where management is limited to restorative procedures or tooth extraction. A surgeon general review in 2000 underscored the links between oral health and systemic health and emphasized persistent inequalities in access to proper dental care for all age, ethnic, and socioeconomic groups [1]. Despite decades of preventative efforts, data indicate that little progress has been made and caries remain highly prevalent [2]. In Canada, caries-related conditions account for 4 million lost workdays and 2 million lost school days annually, contributing to an estimated $12.8 billion dental care burden, second only to cardiovascular disorders in health care costs [3]. Globally, tooth decay is the most prevalent of 291 diseases recognized by the World Health Organization, affecting nearly all adults [4]. This highlights the need for scalable adjunct therapies to the existing prophylactic paradigm of brushing and flossing. Some suggested approaches include bioactive remineralization strategies (e.g. fluoride and calcium–phosphate technologies), biofilm-control, non-cariogenic sugar substitutes (notably xylitol, with mixed clinical evidence), and probiotics aimed at shifting the oral microbiome away from acidogenic, cariogenic communities rather than broadly sterilizing plaque [5–7].

Dental caries is now widely understood as a biofilm-mediated disease. The mutans group of *Streptococci* – most notable is *Streptococcus mutans* – are the keystone pathogens that produce exopolysaccharides from dietary sucrose that facilitate tooth adhesion and the formation of acidogenic biofilms (plaque) on the enamel hydroxyapatite [8,9]. Within the biofilms, a diverse microbiome ecology sustains carbohydrate fermentation that generates the localized acidic microenvironment, overcoming the buffering capacity of saliva to shift the community to a cariogenic state that becomes self-reinforcing without intervention. This dysbiotic ecology is further amplified by cross-kingdom interactions, as *Candida albicans* co-aggregates with *S. mutans* to form mixed-species biofilms that enhance biomass accumulation, acidogenicity, and biofilm stability, particularly in early childhood caries (ECC) [10,11]. For these reasons, conventional antimicrobial interventions are often short-lived in the context of a self-reinforcing plaque biofilm that rapidly re-establishes cariogenic dominance. In contrast, approaches that shift microbial ecology by selectively suppressing cariogenic taxa, disrupting biofilm structure, and enabling stable colonization by health-associated microbes, may prolong the therapeutic effects of brushing and flossing by stabilizing a non-cariogenic ecosystem for longer periods. Probiotic strategies designed to integrate into oral biofilms and competitively antagonize *Streptococcus mutans* represent a biologically aligned and potentially more durable approach to caries control [12,13].

Probiotic therapies have been explored as an all-natural means of regulating *S. mutans* and the broader microbiota, with most clinical and preclinical studies focusing on single strains [14–24]. However, the mechanisms of action are usually unspecified or, when studied, found to be highly strain-specific, including co-aggregation with *S. mutans,* the secretion of antimicrobial bacteriocins or hydrogen peroxide, and competitive colonization of plaque niches [25–27]. This mechanistic diversity suggests that combining strains with complementary activities may yield synergistic suppression of cariogenic biofilms compared to single strains alone. We therefore hypothesized that consortia of strains with anti-*S. mutans* effects may act synergistically to inhibit or reduce the formation of pathogenic biofilms in a clinically relevant *in vitro* environment.

Thus, we set out to systematically identify the relative synergistic capacity of probiotic strains. We first screened individual candidate probiotic strains for their anti-caries properties, including *S. mutans* antagonism, hydrogen peroxide production, and hydroxyapatite adhesion. We then assessed strains with anti-caries properties for multi-strain synergistic antagonistic effects against *S. mutans* isolates. Overall, the results illustrated that the combination of *Lactiplantibacillus plantarum* (formerly *Lactobacillus plantarum*) and *Lactobacillus helveticus* strains displays synergistic *S. mutans* antagonism, with strains *L. plantarum* Stellar™ Lp-199 and *L. helveticus* Stellar™ Lh-170 exhibiting the greatest combined potency. This combination was further evaluated for *in vitro S. mutans* and *C. albicans* biofilm preventive and disruptive abilities in comparison to *S. salivarius* DSM 14865 as a benchmark anti-caries strain [22]. Validation of the findings through *in vitro* biofilm models suggests that the consortia could be an effective microbiological adjuvant for prophylactic caries management.

## Materials and methods

### Cells, media, and growth conditions

Stocks of all lactobacilli and bifidobacteria were maintained on de Man-Rogosa-Sharpe (MRS; Difco) agar. Strains of *S. mutans* (including ATCC 25175 – a strain isolated from carious dentin) were maintained on brain‒heart infusion with 1% yeast extract (BHYE; Difco) agar. *Streptococcus salivarius* and *Streptococcus thermophilus* strains were maintained on CABK 12 agar [28]. *C. albicans* TIMM 1768, a strain known to induce oral candidiasis, was maintained on Sabouraud dextrose agar (SDA; Difco). Overnight broth cultures of all probiotics and *C. albicans* were prepared in YEPD (1% yeast extract; 2% peptone; 2% dextrose). Overnight cultures of *S. mutans* were prepared in BHYE. All of the above strains were cultured for 18–22 h under microaerophilic conditions (37°C, 5% CO_2_). Selective plating for *S. mutans* was carried out on mitis salivarius-bacitracin (0.2 U/ml) agar (MSB; Difco) supplemented with 20% sucrose, for 48 h under microaerophilic conditions (37°C, 5% CO_2_), as this method was found to reliably differentiate *S. mutans* from other streptococci and enrich the naturally harsh MSB medium. To mimic the physiological conditions of the oral cavity, hydroxyapatite adhesion assays and all biofilm assays were performed in basal medium mucin (BMM) artificial saliva prepared according to Wong and Sissons (2.5 g partially purified pig gastric mucin, 5 g yeast extract, 5 g protease peptone, 2.5  mg haemin, 1 mg menadione, 33.5  mmol KCl, 1 mmol arginine, 1 mmol urea, mixed in 1 L of Milli-Q water and autoclaved) [29]. For biofilm prevention and disruption assays, BMM was supplemented with 2% sucrose and 1% fetal bovine serum (Gibco), as these ratios promoted the most robust biofilm formation by *S. mutans* and *C. albicans*.

Dental plaque from two consenting adults was collected using sterile probes, spread directly onto MSB + 20% sucrose agar, and incubated for 48 hours at 37°C, 5% CO_2_. Individual colonies were then quadrant streaked onto BHYE agar and incubated again for 24 h at 37°C, 5% CO_2_. Genotyping was undertaken on the isolated colonies to confirm their species. Bacterial DNA was extracted using InstaGene Matrix (BioRad) according to the manufacturer’s instructions. All PCR reagents were obtained from Invitrogen. A total of 30  ng of DNA template was added to a master mix of 15  mM MgCl_2_, 200  mM dNTPs, 10  µM MUT-F primer, 10  µM MUT-R primer and 5 U of Taq DNA polymerase in 1X PCR buffer for a 50 µL total reaction volume. The genotyping primers used were MUT-F 5′-ACTACACTTTCGGGTGGCTTGG-3′ and MUT-R 5′-CAGTATAAGCGCCAGTTTCATC-3′, specific to a 517-bp DNA region of the gtfB extracellular glucosyltransferase of *S. mutans* [30]. The thermocycling conditions were as follows: 95°C for 1  min; cycling 95°C for 30 s, 59°C for 30 s, 72°C for 1  min for 30 cycles, followed by 72°C for 5  min. The products were separated on a 1.5% agarose gel with 0.05% ethidium bromide.

### Hydrogen peroxide production assay

To test the candidate probiotics for hydrogen peroxide production, a methodology similar to Kang et al. was employed [31]. Standard agar growth media (MRS, BHYE, CABK12) were modified by the addition of 0.25  mg/mL 3,3',5,5'-tetramethylbenzidine (TMB) and 0.1  ng/mL peroxidase. TMB was first dissolved in dimethyl sulfoxide (DMSO) (0.125 g/mL) and peroxidase in water (5 mg/mL), then filter sterilized and added to autoclaved liquid agar before plate preparation. Single colonies were streaked directly onto TMB/peroxidase and standard MRS/BHYE/CABK12 control plates before incubation for 72 h at 37°C, 5% CO_2_. The colonies and surrounding agar were assessed for color every 24 h and compared to control plates. A total of three biological replicates were completed.

### Measurement of deferred antagonistic activity

Deferred antagonism assays were performed as previously described by Tagg and Bannister [32]. Overnight suspensions, or 1:1 mixtures of overnight suspensions of Lactobacilli, Bifidobacteria, and Streptococci probiotics, were swabbed onto brain heart infusion (BHI; Difco) plates supplemented with 0.1% (w/v) CaCO_3_ in a measured 1 cm wide edge-to-edge streak using sterile cotton swabs. The plates were incubated for 48 h at 37°C, 5% CO_2_. Afterward, bacterial growth was removed from the surface with a glass microscope slide, and the plates were sterilized with chloroform vapor for 20 mins. Overnight suspensions of *S. mutans* were then swabbed perpendicular to the original growth streak, before incubating at 37°C, 5% CO_2_ for 48 h. The zone of inhibition (ZOI) was calculated as the sum of the distances of growth inhibition on either side of the original probiotic streak. One-way ANOVAs followed by Bonferroni post hoc tests were used to compare means. Synergism was calculated as a synergistic quotient (SQ): ZOI of the combination of the strains, divided by the sum of the ZOIs of each individual strain, such that SQ > 1 indicates synergism [33]. The results represent at least four biological replicates for each condition. All statistics throughout were performed using Prism software (GraphPad, v6).

### Hydroxyapatite adhesion assay

Overnight suspensions of probiotics and *S. mutans* were resuspended to 1 × 10^6^ CFU/ml in BMM. 2 mL of each suspension was added to independent wells of a 24-well polystyrene plate in triplicate, and sterile hydroxyapatite discs measuring 7 mm diameter × 1.8 mm thickness were placed in the center of each well (Clarkson Chromatography Products; Pennsylvania, USA). The plates were incubated at 37 °C, 5% CO_2_ for either 6 h or 24 h before the discs were carefully removed and dipped twice in 500 µL of phosphate-buffered saline (PBS; Gibco) to remove non-adherent cells. Discs were then dropped in 1  mL of PBS in a 1.5  mL Eppendorf tube, scraped with a cell-scraper, and sonicated for 15 minutes before enumeration by ‘drop plating’: 10-fold serial dilutions were made, and three 10 µL drops of each dilution were pipetted onto the appropriate agar for each bacterium before incubating for 24 h at 37°C, 5% CO_2_. The dilutions that yielded between 5 and 50 colonies were counted, triplicates were averaged, and CFU per disc was determined. Statistical comparisons between groups were made using a two-way ANOVA with Tukey’s multiple comparison.

### Inhibition of biofilm formation and pathogen viability in vitro

Overnight cultures of pathogens (*S. mutans* and *C. albicans*) were resuspended to concentrations of 1 × 10^7^ CFU/mL in the BMM and probiotics to 1 × 10^8^ CFU/mL in the BMM. Regardless of test group, the concentrations of pathogens and probiotics per well were held constant: 1 × 10^6^ CFU total pathogens and 1 × 10^7^ CFU total probiotics per well of a 96-well polystyrene plate, with a constant 200 µL volume. Thus, for assessing the prevention of mono-species *S. mutans* biofilms, 100 µL of *S. mutans* was added, and for dual-species *S. mutans*/*C. albicans* biofilms, 50 µL of each was added. Likewise, 100 µL of each probiotic was inoculated per well for tests of individual strains, and when Lh-170 and Lp-199 were combined, 50 µL of each was added. When assessing the dose-dependent effects, pathogens were inoculated at 10 [6] total CFU, while total probiotic CFUs were administered in 10-fold dilutions from 10 [6] to 10 [3] per well. All test groups were prepared in duplicate. Following incubation for 24 h at 37°C, 5% CO_2_, the supernatant was carefully removed without disturbing the biofilm. The biofilms were gently washed twice with 200 µL of PBS to remove non-adherent cells, and then 200  µL of fresh BMM was applied before incubating the plates again for 24 h at 37 °C, 5% CO_2_. Finally, the supernatant was removed, and the biofilms were gently washed twice with 200 µL of PBS before biofilm prevention was assessed in one of two ways:

a) Crystal violet staining as per Abe et al. (1994) [34] – biofilms were fixed with 70% ethanol, stained with 0.5% (w/v) crystal violet, and OD_600_ readings were taken. The results represent the means ± SEM of three biological replicates with three technical replicates each. Statistical comparisons between treatment groups and untreated controls were made using a two-way ANOVA with Tukey’s multiple comparison test.
b) Drop plating on selective media – a final 250 µL of PBS was added to each well. Cell scrapers were used to detach biofilms from the wells, and a wide-bore P1000 pipette was used to transfer all material to labeled 1.5 mL Eppendorf tubes before sonicating for 15 min. Ten-fold serial dilutions of the sonicated samples were prepared in PBS before plating on selective media (MSB + 20% sucrose for *S. mutans*; SDA for *C. albicans*), and incubating for 24 h at 37°C, 5% CO_2_. Enumerations of two technical replicates were used to calculate the mean viable *S. mutans* or *C. albicans* CFUs from the biofilms within each biological replicate. The results represent the mean ± SEM of at least three biological replicates. Statistical comparisons between treatment groups and untreated controls were made using a two-way ANOVA with Tukey’s multiple comparison test.

### Disruption of pre-formed S. mutans biofilms in culture

Similar to above, 1 × 10^6^ CFU total of pathogens (*S. mutans* alone or in combination with *C. albicans*) were inoculated into the wells of a 96-well polystyrene plate, with a total volume of 200 µL of BMM. The plates were incubated for 24 h at 37°C, 5% CO_2_ to allow biofilms to establish, before the supernatant was carefully removed. The biofilms were gently washed twice with 200 µL of PBS to remove non-adherent cells. A total of 1 × 10^7^ CFUs of probiotics (DSM 14865 or Lh-170 and Lp-199) suspended in 200 µL of BMM, were added to each well except the untreated control (200 µL of BMM only). All test groups were prepared in duplicate. The samples were incubated at 37°C, 5% CO_2_ for 10  min, 6  h, 9  h, 12  h, 24  h, 36  h and 48  h to assess the rate of biofilm disruption. At each endpoint, the samples were washed twice with 200 µL of PBS to remove non-adherent cells and resuspended in 250 µL of fresh PBS. Cell scrapers were used to detach biofilms from the wells, and a wide-bore P1000 pipette was used to transfer all material to labeled 1.5 mL Eppendorf tubes before sonicating for 15 min. Ten-fold serial dilutions of the sonicated samples were prepared in PBS before plating on selective media. Enumerations of two technical replicates were used to calculate the mean viable *S. mutans* or *C. albicans* CFUs from the biofilms within each biological replicate. Drop plating on MRS was used to evaluate the ability of the Lh-170 and Lp-199 combination to penetrate the pre-formed biofilms. The results represent the means ± SEM of at least three biological replicates. Statistical comparisons between treatment groups and untreated controls were made using a two-way ANOVA with Dunnett’s multiple comparison test.

### Disruption of pre-formed S. mutans biofilms on extracted human molars

Extracted tooth specimens (permanent 1^st^ and 2^nd^ molars) were obtained from the Interface Center for Oral and Maxillofacial Surgery (London, Ontario, Canada) after approval by the Fanshawe College Research Ethics Board (Protocol: 17-02-02-1). The teeth were cleaned of excess tissue via mechanical scraping, sectioned into halves of approximately equal size with a diamond bur, and submerged in 13% sodium hypochlorite for 24 h. Each half was rinsed twice in 5 ml of sterile PBS and then stored in fresh PBS until used.

One-half of a molar was incubated with 1 × 10^6^ CFU of *S. mutans* in 6  ml of BMM for 24 h at 37°C, 5% CO_2_, on a rotary stand set to ~100 rpm to promote biofilm formation. Using sterile forceps, each tooth was then gently dipped in 6 mL of PBS twice to wash away non-adherent cells. A total of 1 × 10^7^ CFUs of probiotics (DSM 14865 or Lh-170 and Lp-199) suspended in 6 mL of BMM were added to each tooth sample except the untreated control (6 mL of BMM only). The samples were incubated at 37°C, 5% CO_2_ on a rotary stand for 10  min, 6  h, 9  h, 12  h, 24  h, 36  h, and 48  h to assess the rate of biofilm disruption. At each endpoint, samples were gently dipped in 6 mL of PBS twice to remove non-adherent cells, then transferred to a 15 mL Falcon tube containing 6 mL of PBS, where they were sonicated for 30 min. Ten-fold serial dilutions of the sonicated samples were prepared in PBS before plating on selective media. Enumerations of two technical replicates were used to calculate the mean viable *S. mutans* CFUs from the biofilms within each biological replicate. Drop plating on MRS was used to evaluate the ability of the Lh-170 and Lp-199 combination to penetrate the pre-formed biofilms. The final results represent the means ± SEM of at least three biological replicates. Statistical comparisons between treatment groups and untreated controls were made using a two-way ANOVA with Dunnett’s multiple comparison test.

## Results

### Specific strains of L. helveticus and L. plantarum are highly synergistic in their secreted antagonism of S. mutans

Since hydrogen peroxide is a common interbacterial antagonistic metabolite, and *S. mutans* is susceptible to oxidative stress, we screened candidate probiotic strains for its production as a potential anti-cariogenic mechanism. Most *Lactobacillus* strains (10 of 11), both *Streptococcus thermophilus* strains, and all tested *Bifidobacterium* strains produced detectable hydrogen peroxide, whereas *S. salivarius* strains did not (Table 1).

**Table 1.** Hydrogen peroxide production by probiotic strain.

Production within 24 hours	Production within 48 hours	Production within 72 hours	No production by 72 hours
*S. thermophilus* R0083	*L. acidophilus* La-14 *L. reuteri* Lru-1038 *L. salivarius* Ls-33	*S. thermophilus* St-21 *L. rhamnosus* GG *L. plantarum* Stellar Lp-185 *L. rhamnosus* Lr-32 *L. casei* Lc-11 *B. lactis* Bl-04 *B. breve* Bb-03 *B. bifidum* R0071	*S. salivarius* BAA-1024 *S. salivarius* DSM 14865 *L. paracasei* Lpc-37
*L. plantarum* Stellar Lp-199			
*L. helveticus* Stellar Lh-172			
*L. helveticus* Stellar Lh-170			
*B. longum* R0175			

S. mutans ATCC 25175 was used as the initial benchmark for qualifying deferred antagonism. Only five strains (*S. salivarius* BAA-1024, *S. salivarius* DSM 14865, *L. plantarum* Stellar Lp-199, *L. helveticus* Stellar™ Lh-172, and *L. helveticus* Stellar^TM^ Lh-170) from Table 1 produced any inhibitory effects on ATCC 25175 within a 1 cm wide streak (Supplemental Table 1) and were therefore investigated for quantitative synergies against ATCC 25175 and four other *S. mutans* isolates by measuring the additive ZOIs external to the 1 cm streak in all possible two-strain combinations (Table 2). In general, *S. mutans* ATCC 25175 was more resistant to antagonistic factors secreted by the probiotics compared to fresh isolates – no individual probiotics displayed *S. mutans* ATCC 25175 inhibition adjacent to the 1 cm-wide probiotic streak, and few combinations did, whereas each isolate was inhibited by at least 3 individual probiotics and multiple probiotic combinations (Table 2). As single strains, *L. helveticus* Stellar Lh-170 and *L. helveticus* Stellar Lh-172 displayed the widest range of inhibition.

**Table 2.** Determination of synergistic deferred antagonism against five isolates of ***S. mutans.***

	*S. mutans* 25175			*S. mutans* 1			*S. mutans* 2			*S. mutans* 3			*S. mutans* 4	
Strain or Combination	ZOI (mm)	SQ		ZOI (mm)	SQ		ZOI (mm)	SQ		ZOI (mm)	SQ		ZOI (mm)	SQ
BAA-1024	0.00	N/A		11.5	N/A		6.50	N/A		0.00	N/A		1.5	N/A
DSM 14865	0.00	N/A		0.75	N/A		0.00	N/A		0.75	N/A		0.00	N/A
Stellar Lp-199	0.00	N/A		1.14	N/A		1.14	N/A		3.57	N/A		5.71	N/A
Stellar Lh-172	0.00	N/A		1.67	N/A		5.33	N/A		6.67	N/A		5.67	N/A
Stellar Lh-170	0.00	N/A		4.71	N/A		4.00	N/A		1.00	N/A		0.00	N/A
BAA-1024 + DSM 14865	0.00	0.00		1.00	0.08		0.50	0.08		1.25	1.67		0.00	0.00
BAA-1024 + Stellar Lh-170	0.00	0.00		6.75	0.42		1.00	0.10		4.50	1.26		0.00	0.00
BAA-1024 + Stellar Lh-172	0.00	0.00		3.25	0.25		0.00	0.00		2.75	0.41		0.00	0.00
BAA-1024 + Stellar Lp-199	0.00	0.00		3.00	0.24		1.50	0.20		2.67	2.67		0.00	0.00
DSM 14865 + Stellar Lh-170	0.00	0.00		5.00	0.92		6.00	1.50		9.50	2.20		2.50	0.44
DSM 14865 + Stellar Lh-172	0.00	0.00		4.25	1.76		3.25	0.61		4.50	0.61		0.00	0.00
DSM 14865 + Stellar Lp-199	5.75	∞		11.00	5.81		10.50	9.19		10.25	5.86		8.50	∞
Stellar Lh-170 + Stellar Lh-172	0.00	0.00		6.50	1.02		1.75	0.19		5.00	0.49		4.25	0.37
Stellar Lh-170 + Stellar Lp-199	13.00‡	∞		20.14‡	3.44		20.29‡	3.94		19.29‡	4.22		23.86‡	4.18
Stellar Lh-172 + Stellar Lp-199	0.00	0.00		9.25†	3.29		9.75*	1.51		13.75†	1.79		9.25*	1.63

* = *p*<0.05; † = *p*<0.01; ‡ = *p*<0.001. SQ = synergistic coefficient (probiotic combination ZOI / sum of individual probiotic ZOIs). N/A = not applicable. Note: ZOIs are measured externally to the initial 1-cm-wide streak of probiotics.

The average ZOIs were often greater when two probiotic strains were combined. Together, *L. helveticus* Stellar Lh-170 and *L. plantarum* Stellar Lp-199 resulted in the largest ZOIs against all five strains of *S. mutans*. The ZOIs of this combination were significantly larger than the sum of the individual ZOIs against all *S. mutans* strains (*p* < 0.001), indicating synergistic inhibition (Table 2). The synergistic quotients were calculated to be  ∞ , 3.44, 3.94, 4.22 and 4.18 against *S. mutans* ATCC 25175, and isolates 1–4, respectively, where a quotient greater than 1 indicates a level of combined antagonistic activity that is greater than the sum of each probiotic’s individual antagonistic activity. The pairing of *L. plantarum* Stellar Lp-199 with *L. helveticus* Stellar Lh-172 also resulted in synergism against all isolates (*p* < 0.05); and with *S. salivarius* DSM 14865 resulted in non-significant synergism against all five *S. mutans* strains. However, neither of these combinations displayed ZOIs as large as the *L. helveticus* Stellar Lh-170 and *L. plantarum* Stellar Lp-199 combination.

### Select strains adhere to hydroxyapatite as a model of the tooth surface

Durable prophylactic antagonism is contingent on probiotic localization with *S. mutans* at tooth surfaces, the primary site of cariogenic biofilm formation. Thus, we evaluated the ability of *L. helveticus* Stellar Lh-170 and *L. plantarum* Stellar Lp-199 to adhere to hydroxyapatite in the BMM, in comparison to *S. salivarius* DSM 14865 and *S. mutans* 25175 (Figure 1). After 6 h, *L. helveticus* Stellar Lh-170 displayed the weakest adhesion (1.2 × 10^5^ CFU/disc), and *S. mutans* displayed the strongest (5.9 × 10^5^ CFU/disc); however, none of the differences were significant. After 24 h, all bacteria trended towards increased adherence relative to that at 6 h, but only *L. plantarum* Stellar Lp-199 was significantly increased (*p* < 0.001), with values of 1.06 × 10^7^ versus 3.9 × 10^5^ CFU/disc. *L. plantarum* Stellar Lp-199 also displayed significantly increased adherence relative to all other tested bacteria, including S. mutans, at 24 hours (*p* < 0.001).

**Figure 1.**
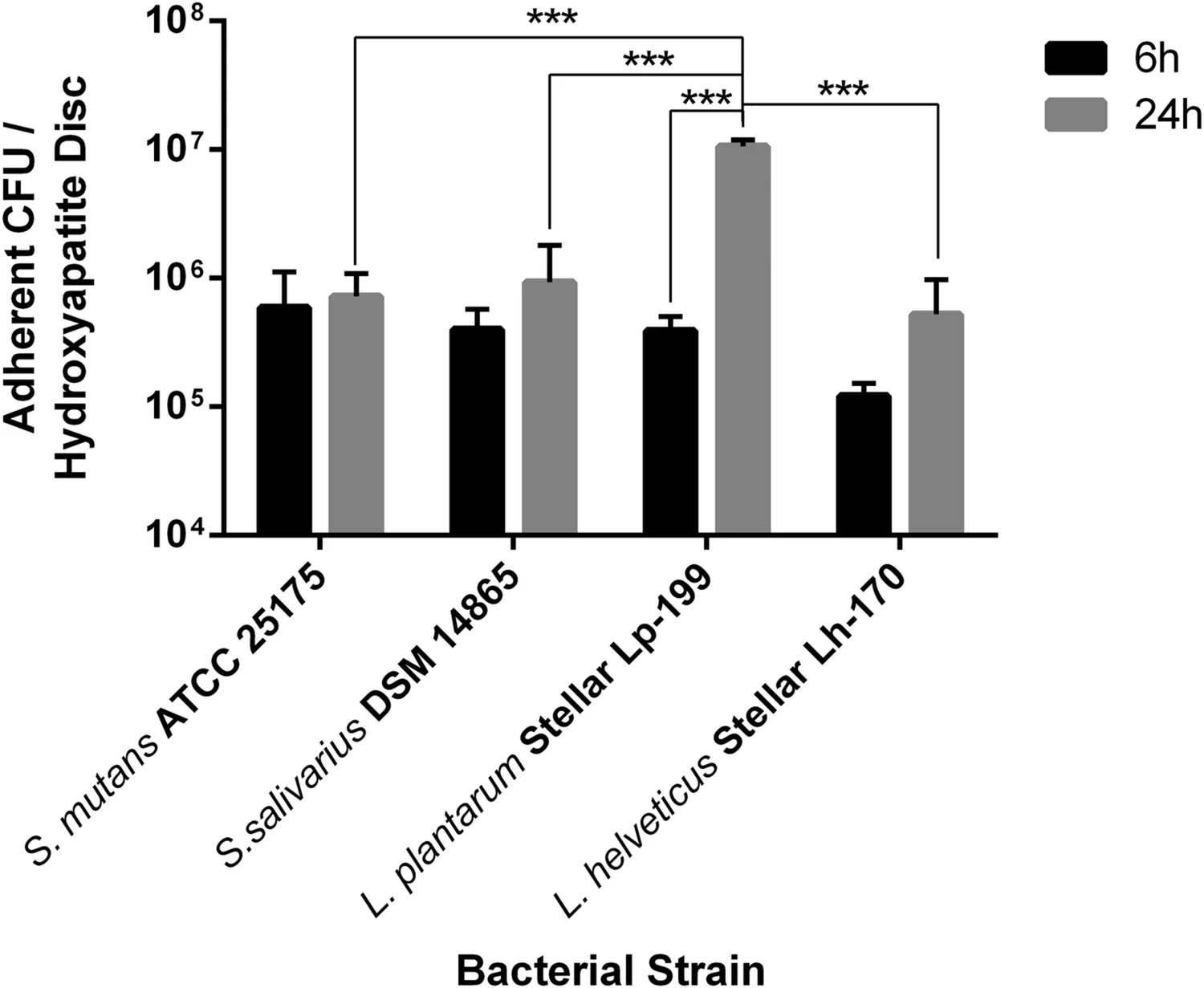
**Adhesion to hydroxyapatite.** 2x10^6^ CFU of each bacterium was inoculated onto a 7mm x 1.8mm hydroxyapatite disc in a 24-well plate and incubated for 6 or 24 h. Non-adherent cells were removed and adherent cells were quantified by drop plating. The results represent the means ± SEM of three biological replicates with three technical replicates each (*** = *p* < 0.001).

### L. helveticus Stellar Lh-170 and L. plantarum Stellar Lp-199 prevent biofilm formation

Given that *S. mutans’* role as a key in biofilm generation, and our demonstrated synergistic antagonism of *S. mutans* by the *L. helveticus* Stellar Lh-170 and *L. plantarum* Stellar Lp-199 combination, we sought to evaluate their joint ability to prevent *S. mutans* biofilm formation compared to *S. salivarius* DSM 14865 as a benchmark anti-caries strain [22]. Using crystal violet staining, both Lh-170 and the Lh-170/Lp-199 combination reduced biofilm formation by approximately 62%, whereas Lp-199 reduced biofilm formation by 25% and that of DSM 14865 by only 5% (Figure 2A), but none of the group’s differences were statistically significant from each other or the control. Using selective plating for *S. mutans*, all the groups significantly reduced *S. mutans* viability relative to the control by at least 99% (*p* < 0.05; Figure 2B). No treatment group was significantly better than another. However, no viable *S. mutans* cells were recoverable after co-incubation with the Lh-170/Lp-199 combination, representing a > 10,000,000-fold reduction.

**Figure 2.**
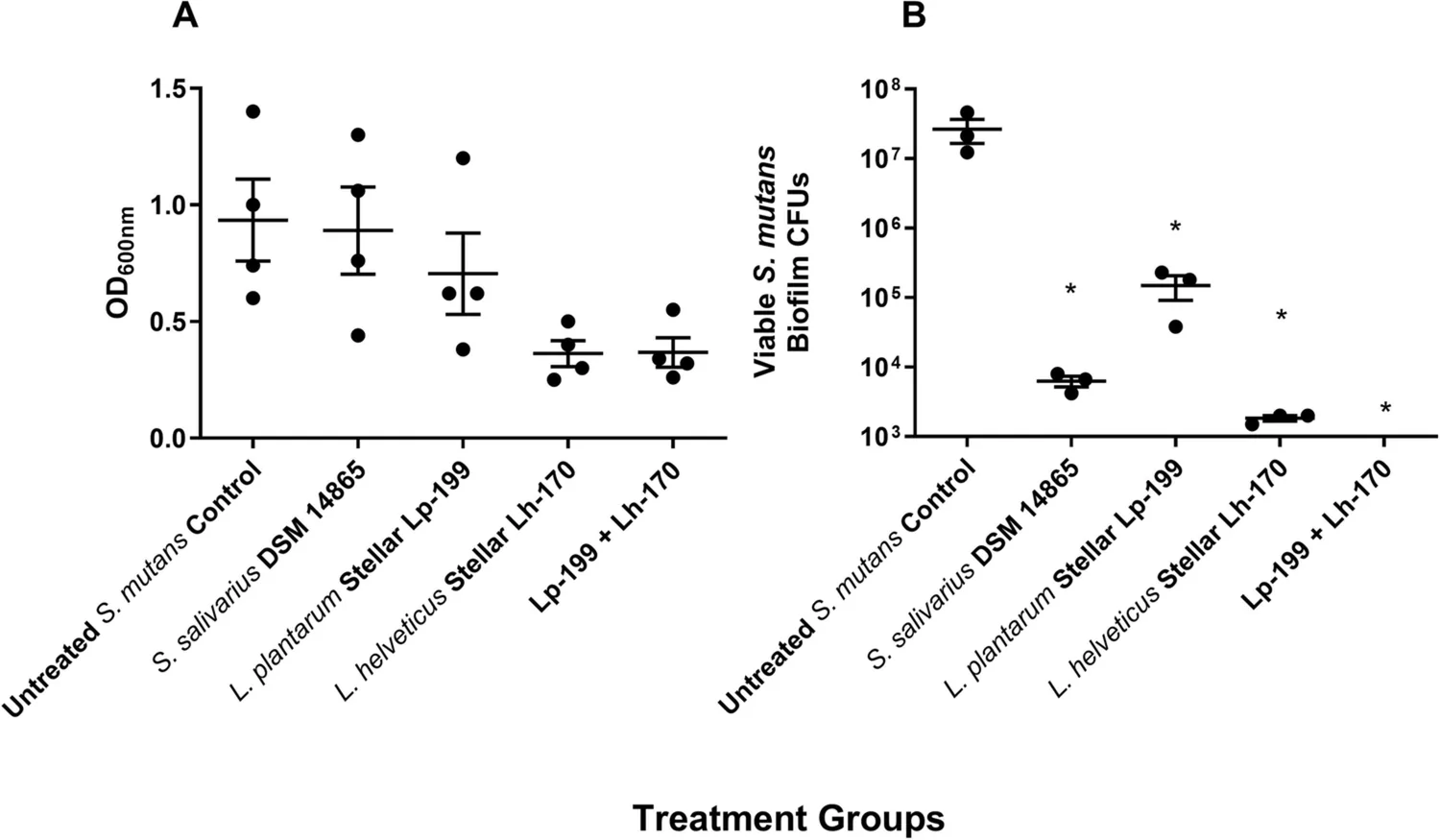
Inhibition of *S. mutans* biofilm formation by probiotics. 10^6^ CFU of *S. mutans* and 10^7^ total CFU of probiotics were co-incubated for 24 h on 96-well polystyrene plates. Non-adherent cells were removed, and adherent cells were quantified by: (A) crystal violet staining; (B) selective drop plating for *S. mutans*. The results represent the means ± SEM of at least three biological replicates. Significant reductions in *S. mutans* viability were observed for all treatment groups compared to the control (* = *p* < 0.05).

Recently, analysis of dental plaques from children with dental caries revealed that *S. mutans* and *C. albicans* commonly form mixed-species biofilms, and that the co-occurrence of these pathogens may increase susceptibility to dental caries [11,35]. Previous research by our group has revealed the ability of probiotics to prevent *C. albicans* biofilm formation [36]. Thus, we assessed the ability of *S. salivarius* DSM 14865 and the *L. helveticus* Stellar Lh-170 and *L. plantarum* Stellar Lp-199 combination to prevent mixed-species biofilm formation by *S. mutans* and *C. albicans* when co-incubated (Figure 3A/3C). We also tested the dose-dependency of the effect by progressively decreasing the initial probiotic inoculum by factors of 10 (Figure 3B/3D). At an initial inoculum of 10 [7] CFU – 10x greater than the pathogens – DSM 14865 did not significantly reduce *S. mutans* viability (3.76-fold reduction) but did significantly reduce *C. albicans* viability by 14-fold (*p* < 0.01). Comparatively, the Lh-170/Lp-199 combination reduced *S. mutans* and *C. albicans* viability by 6182-fold and 231-fold, respectively (*p* < 0.01). At a 1:1 ratio with pathogens, the Lh-170/Lp-199 combination still yielded a statistically significant 17.5-fold reduction (*p* < 0.05) in viable *S. mutans.* Interestingly, when the ratio was skewed 10:1 in favor of pathogens, the combination still significantly reduced *S. mutans* viability by 7.7-fold (*p* < 0.05; Figure 3B).

**Figure 3.**
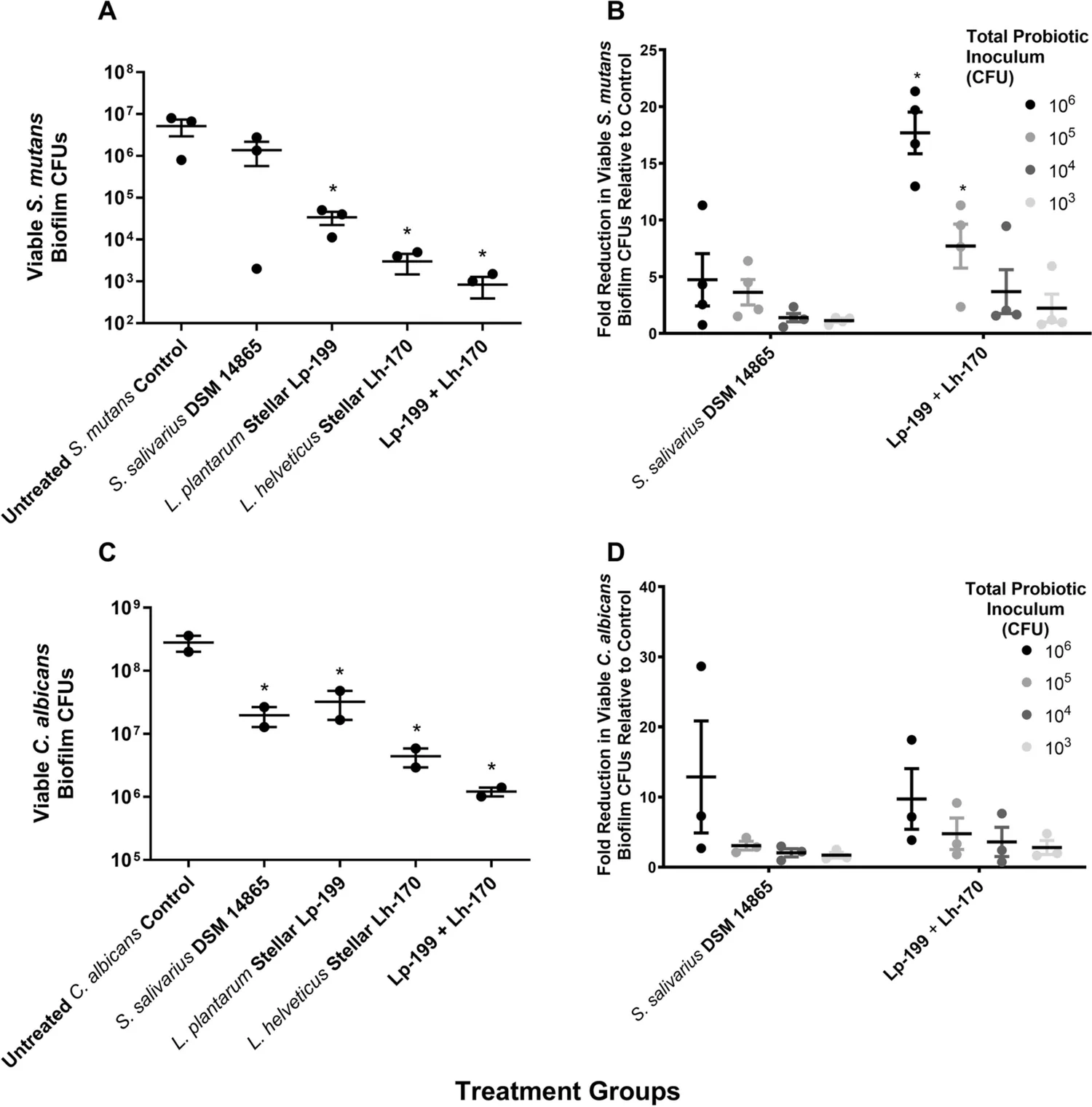
Inhibition of *S. mutans/C. albicans* mixed species biofilm formation by probiotics. 5x10^5^ CFU of *S. mutans* and *C. albicans*, and 10^7^ total CFU of probiotics (A/C) or 10^3^-10^6^ total CFU of probiotics (B/D) were co-incubated for 24 hours on 96-well polystyrene plates. Non-adherent cells were removed, and adherent cells were quantified by selective drop plating for *S. mutans* on MSB agar or *C. albicans* on SDA. Results represent the means ± SEM of at least three biological replicates. The *L. helveticus* Stellar Lh-170 and *L. plantarum* Stellar Lp-199 combination displayed the greatest level of mixed-species biofilm prevention for both *S. mutans* and *C. albicans* at a 10^7^ inoculum (A/C) and still significantly reduced *S. mutans* viability when outnumbered 10:1 by pathogens (B) (* = *p* < 0.05).

### L. helveticus stellar Lh-170 and L. plantarum stellar Lp-199 disrupt pre-formed biofilms both in culture and on human molars

Building on our hydroxyapatite adhesion data and our *in vitro* findings showing that the two-strain probiotic combination best inhibits *S. mutans* biofilms in culture, we sought to directly visualize these combined effects in an *ex vivo* tooth-surface model for translatable relevance. In a one-off demonstration (Supplemental Figure 1), one half of a molar was incubated with either 1 × 10^6^ CFU of *S. mutans* alone, or in combination with 5 × 10^6^CFU of each of *L. helveticus* Stellar Lh-170 and *L. plantarum* Stellar Lp-199 for 24 h. Non-adherent cells were removed, and fresh media were added for an additional 24-hour incubation before SEM imaging to assess biofilm formation. biofilm accumulation on the tooth surface of control teeth, but not probiotic-inoculated teeth. The apparent inhibition was consistent with our culture-based results (Figures 2 and 3).

With apparent near-total *S. mutans* inhibition in culture and exemplified on donor tooth surfaces, and to render this observation into biologically relevant data, we next assessed whether the *L. helveticus* Stellar Lh-170 and *L. plantarum* Stellar Lp-199 combination or *S. salivarius* DSM 14865 harbored any ability to disrupt pre-formed *S. mutans* biofilms. *S. mutans* were inoculated into 96-well plates (1 × 10^6^ CFU) and incubated for 24 hours to establish biofilms. Probiotics were then added to each well (1 × 10^7^ CFU) to assess the efficacy and rate of biofilm disruption over a 48-hour time course. After a 9-hour exposure, the Lh-170/Lp-199 combination significantly reduced the number of viable *S. mutans* biofilm cells by 30-fold (*p* < 0.01), whereas DSM 14865 yielded a 1.7-fold reduction. After 12 h, the Lh-170/Lp-199 combination significantly reduced the number of viable *S. mutans* biofilm cells by 85-fold (*p* < 0.0001), while DSM 14865 provided a statistically significant 6-fold reduction (*p* < 0.001). After 24 h, no viable *S. mutans* cells were recoverable when treated with the Lh-170/Lp-199 combination (*p* < 0.0001), while DSM 14865 only reduced the number of viable *S. mutans* biofilm cells by 6-fold. DSM 14865 achieved a 41-fold reduction in *S. mutans* viability by 48 h (Figure 4A). Enumeration by drop plating of treated biofilm samples revealed that the Lh-170/Lp-199 combination reduced *S. mutans* cell viability, at least one strain of lactobacilli remained viable and integrated into the pre-existing biofilms (Figure 4B). When a comparable experiment was carried out on extracted human half-molars, similar results were achieved (Figure 4C/D). After 9, 12, 24, 36 and 48 h, the Lh-170/Lp-199 combination significantly reduced the number of viable *S. mutans* biofilm cells by 32-fold (*p* < 0.01), 54-fold (*p* < 0.0001), 3,135-fold (*p* < 0.0001), 131,836-fold (*p* < 0.01), and 140,756-fold (*p* < 0.05), respectively. DSM 14865 provided statistically significant 5-fold (*p* < 0.01), 10-fold (*p* < 0.0001), 9.5-fold (*p* < 0.001), 8-fold (*p* < 0.01), and 57-fold (*p* < 0.05) reductions over the same time periods (Figure 4C). Viable lactobacilli were recovered from biofilms on the teeth (Figure 4D).

**Figure 4.**
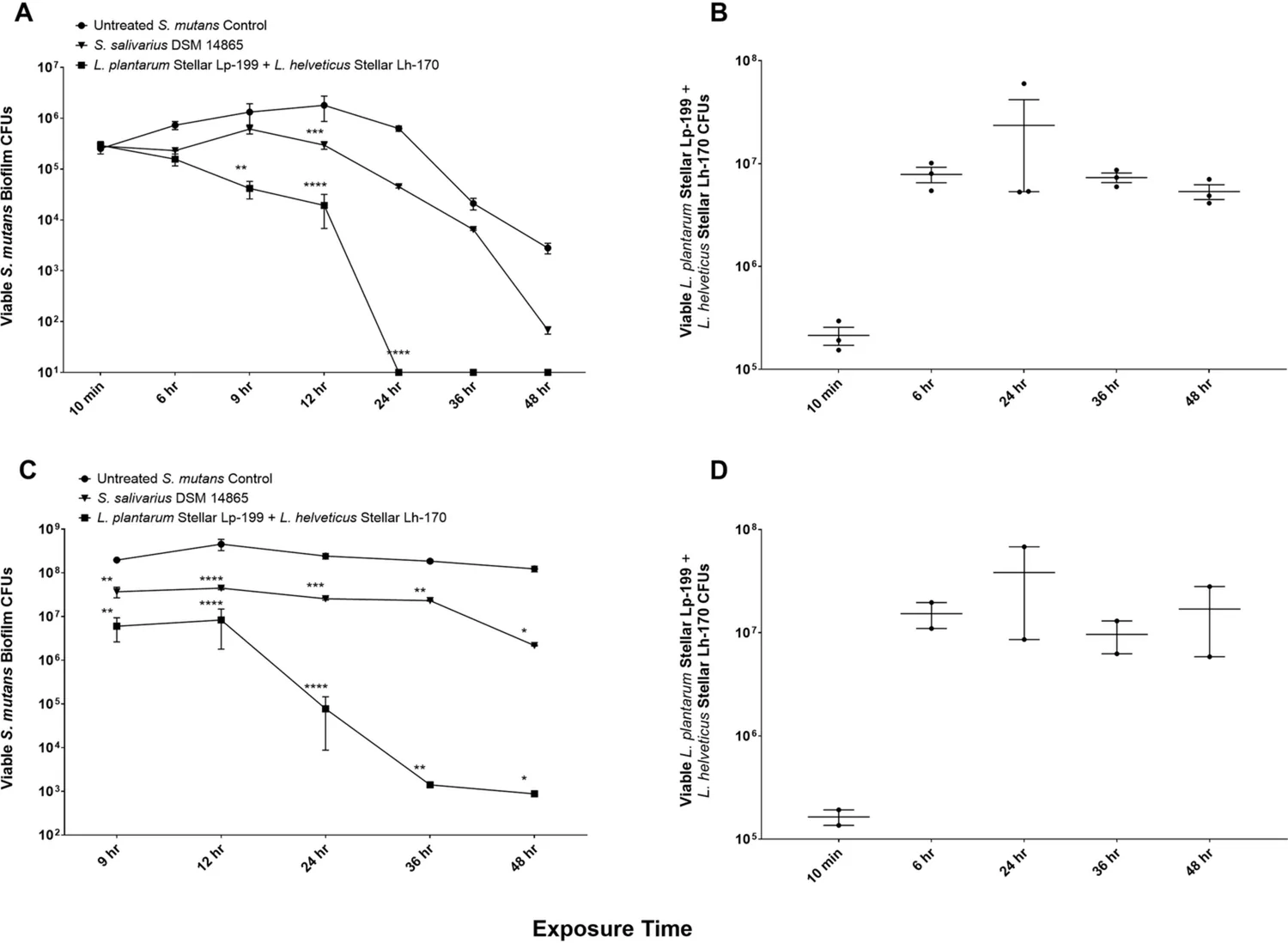
**Disruption of pre-formed** ***S. mutans*** **biofilms by probiotics.** 10^6^ CFU of *S. mutans* were incubated in BMM for 24 hours on 96-well polystyrene plates (A/B) or on one half of a human molar (C/D). The nonadherent cells were removed, and 1 x 10^7^ CFU of probiotics were added to each well before incubating for 10–48 h. Non-adherent cells were removed, and adherent cells were quantified by selective drop plating for *S. mutans* (A/C) or lactobacilli (B/D). The results represent the means ± SEM of at least three biological replicates. The *L. helveticus* Stellar Lh-170 and *L. plantarum* Stellar Lp-199 combination performed better than *S. salivarius* DSM 14865 at disrupting *S. mutans* biofilms on 96-well plates and teeth.

## Discussion

In this study, we demonstrated that combining select probiotic strains can produce synergistic suppression of *Streptococcus mutans* and interfere with the development and persistence of *S. mutans* within cariogenic biofilms *in vitro*. Screening for diffusible antagonism identified several candidate strains with combinations of *L. helveticus* and *L. plantarum* excelling. The specific pairing of *L. helveticus* Stellar Lh-170 with *L. plantarum* Stellar Lp-199 yielded synergistic inhibition of *S. mutans*, exceeding the activity of either strain alone, and outperforming the commonly studied *S. salivarius* DSM 14865 as a benchmark anti-cariogenic strain. Importantly, this combination proved effective in biologically relevant contexts, including adhesion to hydroxyapatite, prevention of *S. mutans* and *C. albicans* biofilm formation, and the disruption of established *S. mutans* biofilms on human molars. The ability of at least one member of this probiotic combination to attach to biofilms while progressively reducing *S. mutans* viability supports a theoretical mechanism in which sustained antagonism is achieved through biofilm localization and continued secretion of inhibitory factors, rather than simply transient antimicrobial exposure. The in vitro antagonistic experiments support a probiotic approach to caries prevention that targets biofilm resilience and microbial community dynamics.

Through various mechanisms, several bacterial strains have demonstrated their abilities to inhibit dental caries-related pathogens. Notably, *S. salivarius* DSM 14865 has been shown both *in vitro* and in clinical studies to express anti-*S. mutans* activity [22]. Additionally, *Lactobacillus rhamnosus* GG has displayed preliminary short-term and long-term anti-dental caries effects [16,37]. Here, we demonstrate that several strains of probiotic bacteria exceed these two probiotics with respect to their *S. mutans* antagonism. *L. rhamnosus* GG did not display antagonism against any of the five strains of *S. mutans* used in this study. Interestingly, several probiotics—including *L. plantarum* Stellar Lp-199 and *S. salivarius* BAA-1024—displayed greater antagonism than DSM 14865 for at least one strain of *S. mutans*, while both *L. helveticus* strains studied (Stellar Lh-170 and Stellar Lh-172) performed with greater or equal antagonism against all five *S. mutans* strains. Compared to all individual probiotics and two-strain combinations, the combination of Lh-170 and Lp-199 yielded the strongest antagonism against all *S. mutans* strains, and the ZOIs were at least two-fold larger than the additive ZOIs of Stellar Lh-170 and Stellar Lp-199 alone. This action is particularly impressive against the *S. mutans* 25175 type strain that is exceptionally pathogenic (having been extracted from a carious lesion) and was not inhibited beyond the initial 1 cm streak by any individual strains (Table 2) [38].

It is unknown what metabolic mechanisms are responsible for the observed synergistic action between Lh-170 and Lp-199. Some strains of *L. helveticus,* secrete bacteriocins such as helveticin J, and helveticin V-1829. Likewise, strains of *L. plantarum* produce a variety of bacteriocins and other antimicrobial substances encoded at the *pln* locus, whose secretion is regulated through a quorum-sensing mechanism [39–41]. Although the production of specific antimicrobial factors by Lp-199 has not been verified, their expression is thought to be relatively common among *L. plantarum* strains [42]. Thus, antimicrobial peptides secreted by these two probiotic strains may form a particularly effective synergistic combination that promotes complementary lethality against various *S. mutans* strains. The non-streptococcal strains that exhibited *S. mutans* antagonism were amongst the strongest H_2_O_2_ producers; however, quick production of H_2_O_2_ was not sufficient to cause antagonism, as *B. longum* R0175 rapidly produced H_2_O_2_ but yielded no *S. mutans* inhibition. H_2_O_2_ is evidently not necessary for antagonism, as *S. salivarius* BAA-1024 and DSM 14865 are H_2_O_2_ non-producers, and therefore may exert their anti-*S. mutans* activity through secretion of specific bacteriocins [43]. Paradoxically, *S. mutans* is able to both produce and degrade H_2_O_2_, but is nevertheless susceptible to it in concentrations above its degradation threshold [44]. Although the mechanism remains unknown, the ability of both Lh-170 and Lp-199 to adhere to hydroxyapatite surfaces, and potentially *S. mutans* biofilms, likely allows these strains to exert their *S. mutans* antagonism in a localized manner.

Not only is biofilm formation on hydroxyapatite a means of increasing antibiotic and immune evasion by *S. mutans,* but it is also essential for dental caries disease pathogenesis. Additionally, recent studies have revealed a relationship between *C. albicans* – the biofilm-forming agent behind oral candidiasis – *S. mutans*, and dental caries biofilm development [10,11,35]. Lactobacilli have been shown to inhibit biofilm formation by both of these species individually [36,45,46]. Thus, we tested the *L. helveticus* Stellar Lh-170 and *L. plantarum* Stellar Lp-199 combination for its ability to prevent *S. mutans* biofilms alone or in combination with *C. albicans*. With respect to *S. mutans* mono-species biofilm formation, the Lh-170 and Lp-199 combination yielded a 62% (n.s.) reduction in biofilm biomass, while significantly reducing the viability of *S. mutans* by ~10,000,000-fold, compared to a 5% (n.s.) reduction in biomass and ~10,000-fold reduction in *S. mutans* viability associated with DSM 14865. In mixed-species biofilms, the Lh-170 and Lp-199 combination resulted in significant > 6000-fold and > 200-fold reductions in *S. mutans* and *C. albicans* viability, respectively, compared to 3.76 (n.s.) and a significant 14-fold reduction by DSM 14865. The reduction in *S. mutans* viability is consistent with some studies that show inhibition when incubated with probiotic candidates, such as *L. plantarum* K41 (98.4% reduction) and *W. cibaria* CMU (~95% reduction) [46,47]. The reduction in *C. albicans* viability is consistent with our previously published data [36].

A strength of our experimental design was the evaluation of probiotic disruption of pre-formed *S. mutans* biofilms in culture and on human teeth *in vitro*. After 24 h, the Lh-170 and Lp-199 combination significantly reduced *S. mutans* viability within pre-formed biofilms by over 10,000-fold on 96-well plates, and by 3,135-fold on teeth. In both experiments, at least one of the lactobacilli was able to adhere and persist in the biofilms, which is congruent with our data that they adhered well to hydroxyapatite. In contrast, DSM 14865 reduced the number of viable *S. mutans* biofilm cells by 6-fold on 96-well plates and 9.5-fold on teeth in the same timeframe. It is well known that quorum sensing in *S. mutans* will naturally dissipate biofilms at high cell densities, limiting the scope and reliability of 96-well plate models, especially at longer timepoints [48]. This could explain the sharp decrease in adherent *S. mutans* cells after 24 h on polystyrene, even in the control group. We posit that our tooth model circumvents this issue, as reflected in the data (Figure 4A vs. 4C), owing to a larger volume of solution and surface area for binding, delaying the effects of quorum sensing on CFU counts. Irrespective of which model of pre-formed biofilms we used, the Lh-170 and Lp-199 combination proved superior to DSM 14865 at reducing *S. mutans* viability.

Clinical studies have already demonstrated mediation of dental health and dental caries risks in children and adults using single-strain probiotic products. Compared to placebo, consumption of milk supplemented with *L. rhamnosus* LB21 and fluoride slowed the decayed-missing-filled-teeth score progression by 75% in children aged 1–5 over a 21-month intervention and yielded additional health benefits, such as decreased incidence of otitis media and decreased need for antibiotic therapy [17]. A similar prevention study in children utilizing milk supplemented with *L. rhamnosus* GG reduced caries incidence and lowered the mutans streptococci counts [16]. Short-term administration of *Bifidobacterium lactis* Bb-12, *Bifidobacterium animalis subsp. lactis* DN-173 010, or *L. reuteri* ATCC 55730, through various delivery systems have also shown reductions in salivary mutans streptococci in adults [14,23,24]. Finally, the treatment of dental caries-active children with lozenges containing *S. salivarius* DSM 14865 reduced *S. mutans* counts and plaque scores [22]. Based on our preliminary *in vitro* findings, we suggest that the *L. helveticus* Stellar Lh-170 and *L. plantarum* Stellar Lp-199 combination may be effective for the management of dental caries-causing microbes, including *S. mutans* and *C. albicans,* and should be evaluated clinically.

Owing to the acidogenic and aciduric nature of lactobacilli, acidification of saliva could negatively contribute to caries progression. A recent double-blind placebo-controlled clinical trial of the effects of a probiotic lozenge containing *L. helveticus* Lh-170, *L. plantarum* Lp-199, and a remineralization agent, on enamel and salivary parameters revealed no significant differences in salivary buffering capacity or volume, but significant increases in the salivary flow rate and pH (*p* < 0.05), and a 32.6% improvement in the DIAGNOdent enamel health scores (*p* < 0.01) after 56 days compared to placebo [49,50]. While we did not measure the effect of probiotics on pH in our in vitro studies, these in vivo results are reassuring that they do not contribute to drastically altered pH nor a cariogenic nature of these specific strains. Salivary microbiological studies would strengthen the clinical evidence for their efficacy and lack of harm.

## Conclusions

Our results indicate that the combination of specific *L. helveticus* and *L. plantarum* strains produces synergistic antagonism against *S. mutans* and *C. albicans,* with the pairing of *L. helveticus* Stellar Lh-170 and *L. plantarum* Stellar Lp-199 yielding the strongest and most consistent effect. Beyond synergistic inhibition of *S. mutans* growth, this combination effectively prevented biofilm formation and disrupted established *S. mutans* biofilms, performing as well or better than *S. salivarius* DSM 14865. Further clinical evaluation of the two-strain combination may clarify its *in vivo* capacity to reduce the cariogenic microbial burden and promote conditions favorable to enamel remineralization.

## Supplementary Material

Supp 1 S mutans Biofilm SEM.jpg
